# Value-based integrated (renal) care: setting a development agenda for research and implementation strategies

**DOI:** 10.1186/s12913-016-1586-0

**Published:** 2016-08-02

**Authors:** Pim P. Valentijn, Claus Biermann, Marc A. Bruijnzeels

**Affiliations:** 1 Department of Health Services Research, Faculty of Health, Medicine and Life Sciences, Maastricht University, Maastricht, The Netherlands; 2 Department Integrated Care University, Essenburgh, Hierden, The Netherlands; 3 Faculty of Social Science, Ruhr University Bochum, Bochum, Germany; 4 Jan van Es Institute, Netherlands Expert Centre Integrated Primary Care, Almere, The Netherlands

**Keywords:** Integrated care, Coordination of care, Organization models, Delivery of care, Nephrology, Review, Quality improvement, Chronic kidney disease, Renal disease, Economics

## Abstract

**Background:**

Integrated care services are considered a vital strategy for improving the Triple Aim values for people with chronic kidney disease. However, a solid scholarly explanation of how to develop, implement and evaluate such value-based integrated renal care services is limited. The aim of this study was to develop a framework to identify the strategies and outcomes for the implementation of value-based integrated renal care.

**Methods:**

First, the theoretical foundations of the Rainbow Model of Integrated Care and the Triple Aim were united into one overarching framework through an iterative process of key-informant consultations. Second, a rapid review approach was conducted to identify the published research on integrated renal care, and the Cochrane Library, Medline, Scopus, and Business Source Premier databases were searched for pertinent articles published between 2000 and 2015. Based on the framework, a coding schema was developed to synthesis the included articles.

**Results:**

The overarching framework distinguishes the integrated care domains: 1) type of integration, 2) enablers of integration and the interrelated outcome domains, 3) experience of care, 4) population health and 5) costs. The literature synthesis indicated that integrated renal care implementation strategies have particularly focused on micro clinical processes and physical outcomes, while little emphasis has been placed on meso organisational as well as macro system integration processes. In addition, evidence regarding patients’ perceived outcomes and economic outcomes has been weak.

**Conclusion:**

These results underscore that the future challenge for researchers is to explore which integrated care implementation strategies achieve better health and improved experience of care at a lower cost within a specific context. For this purpose, this study’s framework and evidence synthesis have set a developmental agenda for both integrated renal care practice and research. Accordingly, we plan further work to develop an implementation model for value-based integrated renal services.

**Electronic supplementary material:**

The online version of this article (doi:10.1186/s12913-016-1586-0) contains supplementary material, which is available to authorized users.

## Background

The rising number of people suffering from chronic kidney disease (CKD) is becoming a worldwide public health problem due to its significant economic burden [1–3]. Worldwide, initiatives have focused on creating new integrated service models for people with CKD through increased emphasis on prevention and care coordination [4, 5]. The merits of a more integrated approach are evident given the multiple interrelated factors and illnesses (e.g. diabetes, cardiovascular disease, hypertention, obesity, malnutrition, smoking) that underlie the burden of CKD [5]. Controlling these interrelated problems one-by-one leads to fragmented chains of command, duplicated supervision and training schemes, and multiple transaction costs [6–12]. The challenge is to develop integrated care models that take into account the interrelated physical, social and lifestyle factors that underlie the burden of CKD. Such integrated care models need to achieve a three-part goal referred to as ‘Triple Aim’: 1) continuously improve patients’ experiences of care, 2) improve the health of the general population and 3) reduce the health care costs per capita [13]. Throughout this paper, we refer to *value-based integrated care* (VBIC) as patients’ achieved outcomes and experience of care in combination with the amount of money spent by providing accessible, comprehensive and coordinated services to a targeted population. Although integrated care is considered a key strategy to improve Triple Aim outcomes, there is limited evidence about how to develop and effectively implement integrated models that are organised around the human and population dimensions of health [6, 13–16].

The limited evidence on the impact of integrated care can be attributed to the lack of a theoretical basis [6, 16–19]. A poor theoretical foundation makes it difficult to understand and explain how and why integrated care efforts improve Triple Aim outcomes, thus restraining the opportunities to identify integration mechanisms and features that improve outcomes. This theoretical inconsistency hampers a systematic understanding and poses significant challenges for policymakers, commissioners, managers, professionals and researchers to support the effective deployment and evaluation of the VBIC models in practice [17, 20]. To address this knowledge gap, the Rainbow Model of Integrated Care (RMIC) was developed to grasp the complex multidimensional nature of integrated care [21]. The RMIC provides a theory which underpins how integrated care efforts (clinical, professional, organisational and system) act at different levels (micro, meso and macro) and can be defined from multiple stakeholder perspectives (patients, professionals, managers and policymakers). However, there is a lack of research into how these integrated care mechanisms act as a means for improving the Triple Aim. In addition, there is a lack of evidence regarding the strategies needed to effectively implement such VBIC models in practice. A complex disease like CKD especially requires a more integrative rather than a disease-focused approach to address the true burden and the unmet bio-psycho-socio-spiritual and somatic needs of people with this disease [1, 5].

In this paper, we aim to contribute to a better understanding of the concept of VBIC through a conceptual framework. Our ambition is that this framework might facilitate a cross-disciplinary dialogue among researchers, policymakers, managers and professionals on how to develop and evaluate integrated care for people with complex chronic diseases like CKD. The framework is used as a guide to provide an overview of the integrated care strategies and outcomes used for people CKD. The final section of this paper discusses the key issues to consider when developing and monitoring VBIC in practice.

## Methods

### Developing the framework

In a previous study, a theory-driven, qualitative and mixed-method was used to develop the RMIC [21], a model which describes the concept of integrated care. Subsequently, Delphi studies with an interdisciplinary panel of experts from academia and practice were applied to validate and operationalise the preliminary findings [14, 21]. The results of these studies indicated that further work was needed to also include the Triple Aim outcome domains within the RMIC. Continuing this line of research, for this current study, we synthesised the RMIC with the Triple Aim framework [13] into one overarching conceptual model [22]. The lead authors developed a draft of the framework through an iterative process using face-to-face, teleconference and email discussions. To improve the content validity, two external researchers independently reviewed the framework and provided feedback by email regarding the final synthesis of the domains of the model. Based on these discussions, a revised draft of the RMIC was produced.

### Literature review

The revised RMIC was used to provide an overview of the integrated care strategies and outcomes used in renal care. We principally followed the rapid review approach of Khangura et al. [23], which differs from a traditional systematic review in the sense that it fits to the purpose of the knowledge users’ specific needs in circumstances wherein time and resources are limited. A four-step approach was followed to complete the rapid review. First, the research question that would direct the focus of the literature to be reviewed, assessed and included was developed by a small working group consisting of the lead authors (PV, MB) and two external participants. Following the RMIC, we focused on a broad range of integration interventions for people with CKD at four different levels: 1) system (system integration), 2) organisational (organisational integration), 3) professional (professional integration) and 4) service (clinical integration). Second, a systematic literature search was developed in consultation with the working group. The Cochrane Library, Medline, Scopus, and Business Source Premier databases were searched using the following search terms: “kidney disease,” “integrated service system” and “integrated care.” The detailed search and selection strategy appears in “Additional file 1.” Due to time constraints, we were not able to fully review all the articles found within the Scopus database. Third, titles and abstracts were screened by the first author (PV). Articles had to meet the following criteria to be included: 1) available electronically as a full-text, 2) provide a description of an integrated care intervention or impacts reported for renal care, 3) published within the past 15 years, and 4) written in English. Full-text relevance screening was then performed by PV, and reasons for exclusion were recorded. For completeness, we also systematically reviewed the references of each article that met the inclusion criteria. Fourth, a coding template was created in DistellerSR [24] to synthesise the included studies according to the conceptual framework. For every included article, PV categorised the type of integrated care intervention and outcome domains reported. PV and MB met regularly during the coding process to review coding decisions.

### Ethics

As this study does not involve patients or study subjects, according to the Dutch Medical Research in Human Subjects Act (WMO), this is exempt from ethical approval in The Netherlands.

## Results

In the following section, we outline the conceptual framework for VBIC. The narrative synthesis regarding the literature on integrated renal care that follows is organised according to the domains of the VBIC framework.

### The key domains for VBIC

**Fig. 1:**
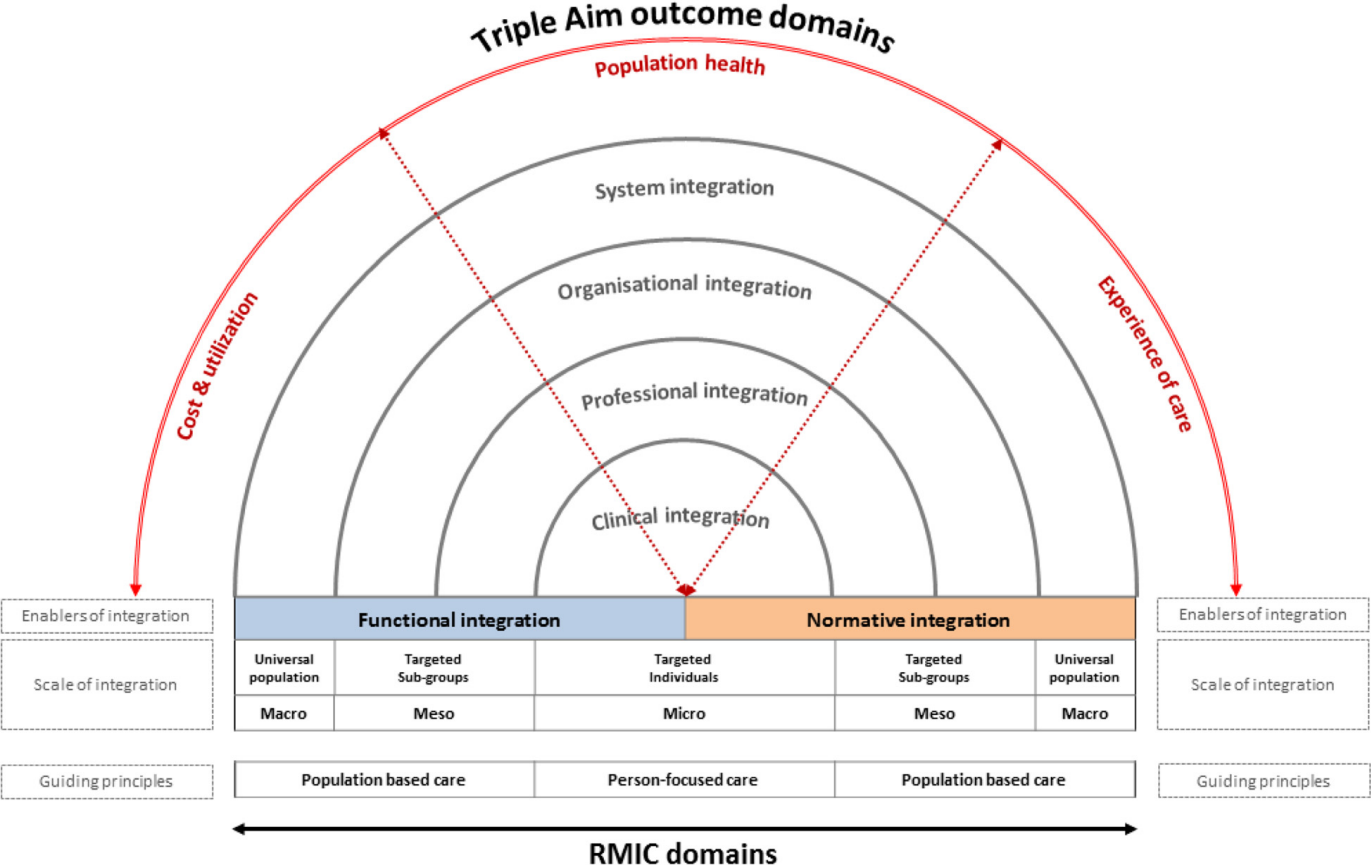
The Rainbow Model of Integrated Care: A multi-perspective on value creation through integration of care. Legend: Schematic representation of value-based integrated care. Adapted from “Rainbow of Chaos: A study into the Theory and Practice” by P.P. Valentijn, 2015, Ede, Print Service Ede. Copyright 2015 by Pim P. Valentijn. Adapted with permission

Figure 1 shows a schematic illustration that combines the RMIC and the Triple Aim framework into one overarching conceptual model. The Triple Aim outcome domains are visualised in the outer bow of the model. The inner bows of the model represent the RMIC, and shows that achieving the Triple Aim requires different types of integration processes. Table 1 lists the key domains of the model that we used to operationalise the concept of VBIC.

**Table 1:** Description of the domains of the revised RMIC

Main domains	Subdomain	Description
Triple Aim Outcomes^a^		
Experience of care	Satisfaction	Patient-reported measures addressing the satisfaction (or barriers) of the service delivery.
	Quality of care^b^	Factors related to the quality of care (e.g. patient safety, timeliness, responsiveness, accessibility).
Population health	Mortality	Health outcomes related to mortality measures for a general or specific (sub)population (e.g. life expectancy, standardized mortality, healthy life expectancy).
	Morbidity	Health outcomes related to patient reported functional status measures (e.g. HRQOL-4, SF-12, EuroQol).
	Disease Burden	Health outcomes related to the incidence and prevalence of (major) chronic conditions (e.g. diabetes, heart diseases, chronic obstructive pulmonary disease).
	Behavioural factors	Health outcomes related to behavioural factors (e.g. smoking, diet and physical activity)
	Physiological factors	Health outcomes related to physiological factors (e.g. body mass index, cholesterol and blood glucose).
Cost and utilization	Cost per capita	Total (direct and indirect) costs and costs by type of service of a particular population per time unit (month, year).
	Utilization of services	Total volume of service use visits (e.g. number of hospital, emergency department) for per a particular population per time unit (month, year).
RMIC domains^c^		
Scale of integration	Universal population (macro)	Universal strategies and interventions designed to promote the general health or reduce the risk of developing health problems in a population.
	Targeted sub-groups (meso)	Targeted strategies and interventions designed for a subpopulations at risk (based on their age, gender, genetic history, condition, or situation) of developing a (severe) disease.
	Targeted individuals (micro)	Targeted strategies and interventions designed for persons at extremely high risk or who already show (a)symptomatic or clinical ‘abnormalities.’
Type of integration	System integration (macro)	Coherent set of (informal and formal) political arrangements to facilitate professionals and organisations to deliver a comprehensive continuum of care for the benefit of the general population.
	Organisational integration (meso)	Inter-organisational partnerships (e.g. agreements, contracting, strategic alliances, knowledge networks, mergers) based on collaborative accountability and shared governance mechanisms, to deliver a comprehensive continuum of care to targeted sub-groups at risk.
	Professional integration (meso)	Inter-professional partnerships based on a shared understanding of competences, roles, responsibilities and accountability to deliver a comprehensive continuum of care to targeted subgroups at risk.
	Clinical integration (micro)	Coordination of person-focused care for a complex need at stake in a single process across time, place and discipline.
Enablers of integration	Functional integration (micro-macro)	Communication mechanisms and tools (i.e. financial, management and information systems) structured around the primary process of service delivery that provide optimal information as a feedback mechanism for decision support between organisations, professional groups and individuals.
	Normative integration (micro-macro)	Mutually respected cultural frame of reference (i.e. shared mission, vision, values and behaviour) between organisations, professional groups and individuals to achieve shared goals towards the Triple Aim outcomes.

^a^Categorization of the Triple Aim domains is based in the IHI’s ‘Guide to measuring the Triple Aim’ [96]

^b^Categorization based on the Institute of Medicine’s six aims for improvement [97]

^c^Categorization of performance domains are based on the RMIC [21]

The integrated care foundation of the model distinguishes two guiding principles: person-focused and population-based; and six integrated care domains: clinical, professional, organisational, system, functional, and normative integration. Both functional and normative integration are conceptualised as enablers to encourage implementation of integrated care across different clinical, professional, organisational and system boundaries. The model visualises that integration efforts can target ‘micro’ (person-focused) as well as ‘meso and macro’ (population-based) dimensions of health. This leads to the recognition that the economies of scale and the scope of the different types of integration are linked to the volumes or risks (prevalence) within a targeted population or subgroup. For example, the prevalence within a targeted population has to be large enough to achieve the quality and efficiency benefits of an organisational integration effort at the meso level [25].

The Triple Aim bow of the model specifies the interdependent endpoints of integrated care in terms of patients’ healthcare costs, population health and experience of care [13]. The model visualises that improving the Triple Aim requires differing integration types across the entire care continuum. Thus, to successfully leverage an integrated care model, it must demonstrate various interdependent patient, social and economic benefits which, in turn, require co-creation and collaboration across all key stakeholders (patients, professionals, managers and policymakers). In order to demonstrate Triple Aim benefits of a particular integrated care effort, it is important to include the cost, health and quality measures across the entire care continuum. This is especially important for patients and populations suffering from complex multiple problems and illnesses which are typically treated by various dispersed professionals, organisations and service systems (e.g. health and social care) across the entire care continuum.

It is essential, however, to keep in mind that environmental hazards as well as lifestyle and social factors have more influence on improving the overall health of complex populations than access to health and care services [7, 26–28]. This means that integrated care services along with medical and economic criteria have to consider person-defined needs and priorities along with addressing the complex illness burden of a defined population, and this consideration is visualised in the Triple Aim and person-focused and population-based domains of the model. The model theoretically underpins that, in order to tailor and prioritize integrated care strategies in an efficient and sustainable matter, integrated care development starts with identifying gaps in outcomes and assessing the service-user needs (instead of the professional requirements) within a targeted, at-risk population. To sum, the revised RMIC distinguishes the integrated care domains: 1) type of integration and 2) enablers of integration and the interrelated outcome domains, 3) experience of care, 4) population health and 5) costs (see Table 1).

### Synthesis integrated renal care

Our search yielded a total of 534 potentially relevant references. Initial screening of titles and abstracts of the original papers resulted in 42 articles for full text screening, of which 26 were included. The reference lists of these included papers were manually screened and 7 additional papers were included, adding up to a total of 33 papers. Most papers were excluded due to their lack of a description of either an integration strategy or the outcomes reported. The majority of the included publications were primary research articles (42 %, *n* = 14); other publications were review articles (33 %, *n* = 11), expert opinion articles (15 %, *n* = 5) and study protocol articles (10 %, *n* = 3). In addition, the majority of the included articles had a moderate (Levels 3 and 4: 82 %, *n* = 28) to weak (level 5: 15 %, *n* = 5) level of evidence. More descriptive information about the included articles can be found in “Additional file 2.” In the following synthesis we describe the reported results for the integrated care domains: 1) type of integration and 2) enablers of integration and the triple aim outcome domains, 3) experience of care, 4) population health and 5) costs reported.

### Type of integrated renal care interventions

Table 2 provides an overview of the integrated care domains reported in the included articles. The majority of articles (*n* = 19) reported a combination of integration interventions which dominantly (*n* = 13) focused on a mixture of clinical and professional integration strategies. For example, peritoneal and home haemodialysis, medication reviews, medical education and multidisciplinary collaboration were described as integrated care efforts. However, organisational integration efforts (e.g. inter-organisational networks and governance arrangements) were hardly (*n* = 2) reported within the integrated renal care literature. Moreover, system integration interventions such as regulatory frameworks and policies were generally not reported at all. Only one article reported indirectly to the needed ‘macro’ system processes for integrated renal care [29]. These findings highlight that integrated renal care services are particularly focused on ‘micro’ operational integration processes, while little emphasis is placed on the ‘meso’ organisational and ‘macro’ system context.

**Table 2:** Integrated care domains reported (*n* = 33)

Scope, n (%)	
Clinical integration	7 (21)
Professional integration	3 (9)
Organisational integration	2 (6)
System integration	na
Combination^a^	19 (58)
Not reported	2 (6)
Enablers, n (%)	
Functional integration	13 (39)
Normative integration	na
Combination^b^	2 (6)
Not reported	18 (55)

^a^Mostly focused on the clinical and professional integration domains

^b^Combination of functional and normative aspects reported

### Enablers of integrated renal care

Table 2 shows that there is a dominant focus (*n* = 13) on functional integration mechanisms within the field of integrated renal care. Most studies reported on functional information technology tools like electronic health records, e-referral systems and information systems to support the communication between patients and/ or providers. These technical (functional) tools mainly focused on the facilitation and coordination of information at the micro level of clinical integration and meso level of professional integration. In contrast, normative integration mechanisms such as mutual trust and respect among different providers where hardly (*n* = 2) (in combination with functional integration mechanisms) reported within the included literature. These findings suggested that the development of integrated renal care services is mainly stimulated through the implementation of technical (functional) enablers.

### Triple Aim domains of integrated renal care

In this section, the Triple Aim domains provide a synthesis of the reported outcome domains within the field of integrated renal care. However, high-quality evidence on the actual Triple Aim outcomes of integrated renal care interventions is generally lacking. Consequently, the reported Triple Aim domains point towards a consensus on the possible impacts of integrated renal care based on clinical experience and expertise, rather than the actual outcomes of integrated renal care interventions. Table 3 provides an overview of the Triple Aim domains reported in the included articles.

**Table 3:** Triple Aim outcome domains reported (*n* = 33)

Experience of care, n (%)	
Satisfaction	1 (3)
Quality of care	13 (39)
Combination^a^	1 (3)
Not reported	19 (58)
Population health, n (%)	
Mortality	1 (3)
Morbidity	3 (9)
Disease burden	1 (3)
Behavioural factors	1 (3)
Physiological factors	6 (18)
Combination^b^	20 (61)
Not reported	1 (3)
Cost and utilization, n (%)	
Cost per capita	3 (9)
Utilization of services	4 (12)
Combination^c^	7 (21)
Not reported	19 (58)

^a^A combination of satisfaction and quality factors reported

^b^Mostly a combination of mortality, morbidity, disease burden and physiological factors

^c^A combination of cost and utilization measures reported

A minority of the included articles (*n* = 2) reported on the experience of care from the patients’ perspective. The articles that reported about the experience of care domain (*n* = 14), mainly reported clinical access and safety measures as patient-related quality indicators (*n* = 13). Missing from the current literature were, however, patients’ reports of satisfaction with care measures. In general, these findings indicated a lack of measures and outcomes for identifying the gaps of care and evaluating the needs of integrated renal care from an end-user perspective.

Contrarily, the population health domain was the most frequently (*n* = 32) reported outcome domain compared to the other two Triple Aim domains. Most articles reported on a combination of the subdomains (*n* = 20), which mainly focused (*n* = 16) on the following subdomains: physiological (e.g. transferrin saturation, blood pressure, calcium, albumin, haemoglobin, HbA1C, LDL-cholesterol), disease burden (e.g. cardiovascular disease, diabetes mellitus, hypertension), morbidity (e.g. EQ5D, KDQOL-36) and mortality (e.g. SMRs). Within this combined population health subdomain most articles (*n* = 12) reported on the positive impact of an integrated renal care intervention in terms of physiological outcomes (see Additional file 2). However, population health outcomes related to the behavioural aspects of health (e.g. smoking, physical activity) were hardly (*n* = 9) reported within combined subdomains (see Additional file 2). These findings point towards the fact that the population health domain within the field of integrated renal care is particularly focused on the physiological dimensions of health (*n* = 20) (see Additional file 2). Meanwhile, the broader psycho-social dimensions of health seem to be neglected within the current literature.

Finally, the vast majority of research on integrated renal care showed a paucity in terms of reported economic outcomes (*n* = 14). When cost outcomes were reported, they were generally (*n* = 11) determined using utilization of care rates (e.g. hospitalisation, ED visits). However, evidence on cost-effectiveness of integrated renal care interventions is scarce and includes several research limitations. For example, when cost and utilization outcomes were reported, the researchers did not provide an overview of all related (direct and indirect) costs across the entire continuum of care. These findings suggested that there is a lack of reporting on cost and utilization measures and their outcomes within the field of integrated renal care.

## Discussion

This study synthesised the theoretical assumptions of the RMIC and the Triple Aim into one overarching framework to specify the concept of VBIC. The framework distinguishes the following integrated care domains: 1) type of integration and 2) enablers of integration and the interrelated outcome domains, 3) experience of care, 4) population health and 5) costs. The different domains provide a crucial differentiation for clarifying and interpreting the mechanisms and the three dimensional value perspectives (patient, social and economic) of integrated care. Based on the framework, a rapid review was conducted to identify the integrated care strategies and outcomes used in renal care. The results showed that integrated renal care interventions particularly focused on ‘micro’ operational integration processes and technical (functional) enablers. Evidence regarding the outcomes of integrated renal care is rather weak and dominantly focused on the physiological dimensions of health. In addition, there is a general lack of measures and outcomes to identify the patient perceived and economic benefits of integrated renal care.

### Contribution of research findings

The revised RMIC presented in this article provides a theory on how integrated care plays complementary roles at the micro level of clinical integration, the meso level of professional and organisational integration, and the macro level of system integration to improve outcomes in terms of patients’ experience of care, population health and costs per capita. Whereas previous models on integrated care tend to focus solely on isolated macro, meso or micro levels of integration [30–32], the revised RMIC highlights the fact that the different levels and perspectives are, in fact, interrelated. In addition, this theoretical analysis also led to the recognition that the value of integrated care can be defined from a patient, social and economic perspective. This multidimensional value perspective contrasts sharply with the traditional mechanistic views of integrated care and value-based care, which promote that standardising the delivery of care leads to better outcomes [33–36]. Existing models tend to overlook the inherent multifaceted social, political and economic factors that influence people’s health and well-being as well as the dynamic complexity of developing integrated care. In addition, industrial quality improvements founded on strategies of cost leadership and differentiation are unachievable when caring for people with complex and multiple problems and illnesses. This kind of logical approach can actually lead to fragmentation. Since the revised RMIC reconfigures the VBIC perspective through the identification of the gaps in care among a targeted, at risk population, integrated services can be better tailored to the end-users’ needs beyond the current unidimensional corporate efficiency approach [37, 38].

The findings of the literature review indicated that, in the field of integrated renal care, there is a prime focus on clinical ‘micro’ integration processes, while the ‘meso’ organisational and ‘macro’ system were generally not considered. These findings are not surprising, given the prime focus of practice, science and policies on the clinical and professional domain of integrated care [6, 14, 15, 39]. However, previous research has highlighted the need to develop a multilayer commitment (e.g. professionals, managers and policymakers) when leading effective integrated care efforts [6, 16, 20, 21, 33, 39–41]. In line with the RMIC, this implies that more emphasis needs to be placed on theorizing, studying and modelling interaction patterns within and between the clinical, professional, organisational and system levels of integrated renal care. Research also has suggested that the barriers to effective integrated care strategies are political rather than technical [13]. This means that ‘soft’ normative (e.g. cultural values) mechanisms are critical enablers for encouraging widespread implementation of integrated care. Previous research has indicated that normative integration mechanisms indeed influence the effective development of integrated care across various political, organisational, professional and clinical fields [42, 43]. However, most studies within the field of integrated renal care tend to focus on ‘hard’ functional aspects (e.g. IT) and have barely taken into account the normative enabling mechanisms. This finding emphasises the need to monitor the normative enabling mechanisms between different professional and organisational groups when developing integrated renal care services. In addition, the present study shows that less emphasis has been placed on ‘macro’ system integration processes. In contrast, research has suggested that political influences are essential preconditions for developing effective integrated health systems [13, 33, 44]. This implies that integrative, rather than disease-specific, policies are needed in order to address the bio-psycho-socio-spiritual and somatic needs of people with CKD. We think further debate about how to develop such integrative policies would be extremely useful.

The integrated renal care evidence synthesis also showed that most of the outcomes reported focused on the physiological dimensions of health. We found this result not surprising given the prime focus of the included literature on the clinical micro processes and related bodily functions. However, there may be a need to revisit our understanding about the definition and operationalisation of the population health domain. Drawing from the new definition of health [45, 46], health is operationalised as a dynamic concept consisting of six dimensions: 1) bodily functions, 2) mental functions and perception, 3) spiritual/existential dimension, 4) quality of life, 5) social and societal participation, and 6) daily functioning. This reconfiguration refers to the ability of people to contribute to their own health through lifestyle, behaviour and self-care, and by optimally adapting professional advice regarding their life circumstances. In this regard, the population health domain of the Triple Aim framework has a dominant focus on the physical and quality of life dimensions of health. This definition of health requires a further reconfiguration of the concept of population health that encompasses life as a whole with more of an emphasis on aspects such as meaningfulness and social participation.

Against this background, including the patient perspective is as important as any organising principle that aims to restructure services around the needs and values of people [6, 47–50]. Notably, only a limited number of integrated renal care studies have attempted to describe or evaluate the experience of care from patients’ perspectives. This lack indicates the need to develop assessment tools and methods to evaluate individual preferences and experiences of care in the field of integrated renal care. Finally, the literature synthesis showed a paucity of research on the economic outcomes of integrated renal care. Consistent with prior findings in the field of integrated care [51, 52], utilization and cost were the most common economic outcomes assessed, although the evidence on cost-effectiveness remains weak. Demonstrating the relationship between economic and health outcomes is generally considered a challenge, because integrated care typically involves multiple changes at multiple levels [33, 51, 52].

### Strengths and weaknesses of this study

It is important to consider the unique strengths and limits of a rapid review. The strength of the present review is that it was theoretically grounded on the RMIC. The revised RMIC has a solid base in the academic literature and expert opinion regarding the concept of VBIC [14, 15, 21]. The present review shows that a theory driven rapid review approach is sufficient to gather and synthesise a broad range of heterogeneous interventions in the literature. The rapid review also identified several potential gaps in the integrated renal care literature consistent with reviews in the general field of integrated care [6, 14, 33, 51, 52].

Due to time constraints, we may have missed some studies from the Scopus database. Moreover, the search was also not complemented by gray literature searches on the Internet. Nevertheless, we did hand search the reference list of the included studies. Another limitation of the rapid evidence approach in this study was that there was only one reviewer involved in the decision making process of including and excluding articles as well as extracting data from the included articles. Although this reviewer was knowledgeable about the content of integrated care and has experience conducting reviews, this limited the scope of the review. We acknowledge that a non-comprehensive evidence synthesis is more prone to bias than a comprehensive synthesis [53]. However, the essential results of the review did not seem to differ extensively from the general field of integrated care [6, 14, 33, 51, 52]. Therefore, we believe that our theory driven rapid review was a reasonable approach towards prioritising a research and development agenda for VBIC renal services.

### Implications for practice and future research

Policymakers, managers, professionals and patients organisations can use the revised RMIC as a guide for developing VBIC in practice. Essential for all key stakeholders is the recognition that the local context matters the most when developing VBIC [13, 54–57]. In other words, the development of VBIC should start with a careful analysis of the needs and system requirements, which can then be used to explore which integration strategy is best suited for whom.

Investment in pioneering research methodologies is necessary in order to reveal the complex interrelationships between the system, organisational, professional and clinical levels of integrated care. The subsequent inference is that research should extend beyond the golden standard of random clinical trials [58] by using evaluation designs that focus on managing complexity by providing ways of monitoring and influencing system state, performance and stakeholders’ behaviour [34, 59, 60]. The main reason for this is that we cannot control all the complexity within a Randomised Control Trial (RCT) design, as blinding and randomisation are impossible within this field. As an alternative to traditional rigid evaluation methods, rapid cycle-evaluations hold much promise for simultaneously evaluating and developing integrated care efforts in an increasingly fast-paced environment [61–63]. Rapid-cycle evaluations can provide timely and actionable evidence as well as reveal possible adaptations to contingencies and, subsequently, help to customize VBIC strategies to local circumstances making them more effective. Future studies should, therefore, operationalise the proposed RMIC toward an analytical and implementation model for VBIC. Such an operationalisation is essential for guiding program implementation, policy formulation and research analysis in the field of VBIC. We plan further work to develop such a model for VBIC renal services, and invite anyone interested in helping to develop and validate the model to contact the authors.

## Conclusion

This study developed a framework to specify the concept of VBIC using the theoretical foundations of the RMIC and the Triple Aim. Based on the framework, a rapid review was conducted to synthesise the current integrated renal care literature. The findings showed that integrated renal care strategies particularly focus on micro clinical processes and physical outcomes, while the evidence regarding strategic impacts is weak. These results underscore that the challenge for the future is to explore which integrated care implementation strategies achieve improved patient health and care experience at a lower cost within a specific context. For this purpose the framework and evidence synthesis has set a developmental agenda for both integrated renal care practice and research.

## Abbreviations

CKD, chronic kidney disease; RCT, randomised control trial; RMIC, rainbow model of integrated care; VBIC, value-based integrated care

## Additional files

Additional file 1: Search and selection strategy. (DOCX 45 kb) Additional file 2: Characteristics of the included articles [29, 64–95]. (DOCX 21 kb)
