# Supplementary material for: Value-based integrated (renal) care: setting a development agenda for research and implementation strategies
Source: BMC Health Serv Res. 2016 Aug 2;16:330. doi: 10.1186/s12913-016-1586-0 (PMC4970292; doi:10.1186/s12913-016-1586-0)

# Additional files

### Additional file 1 – Search and selection strategy

| **Database** | **Search terms** | **Limits** | **Yields** | **Excluded based on title and abstract** | **Full text screening** | **Final inclusion*** |
| --- | --- | --- | --- | --- | --- | --- |
| **Medline** | (("Kidney Diseases"[Mesh]) AND "Delivery of Health Care, Integrated"[Mesh])) | 2000 – 2015 (15 years) | 47 | 28 | 19 | 19 |
|  |  | English |  |  |  |  |
| **Cochrane** | Chronic Kidney Disease and coordination | 2000 – 2015 (15 years) | 2 | 1 | 1 | 1 |
|  |  | English |  |  |  |  |
| **Business Source Elite** | Kidney disease and integrated care | 2000 – 2015 (15 years) | 2 | 2 | 0 | 0 |
|  |  | English |  |  |  |  |
| **Scopus** | Chronic kidney disease and integrated care | 2000 – 2015 (15 years) | 223 | 200 | 23 | 6** |
|  |  | English |  |  |  |  |

* The reference list of the included papers was manually screened and 7 additional papers were included, for a total of 33 papers.

** Due to time constraints, we were not able to fully review and summarise all articles yielded from the Scopus database.


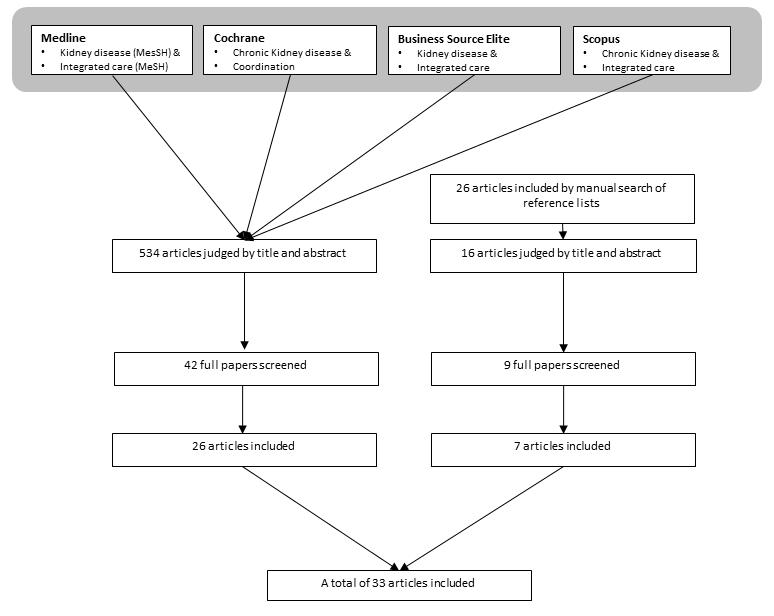

Supplement: Additional file 1: — Search and selection strategy. (DOCX 45 kb) [file 12913_2016_1586_MOESM1_ESM.docx]
