# Supplementary material for: Value-based integrated (renal) care: setting a development agenda for research and implementation strategies
Source: BMC Health Serv Res. 2016 Aug 2;16:330. doi: 10.1186/s12913-016-1586-0 (PMC4970292; doi:10.1186/s12913-016-1586-0)
Supplement: Additional file 2: — Characteristics of the included articles [29, 64–95]. (DOCX 21 kb) [file 12913_2016_1586_MOESM2_ESM.docx]

### Additional file 2 – Characteristics of the included articles

| **Author(s)** | **RMIC domains reported** | | | | | | |  | **Triple Aim outcomes reported** | | | | | | | | | | |
| --- | --- | --- | --- | --- | --- | --- | --- | --- | --- | --- | --- | --- | --- | --- | --- | --- | --- | --- | --- |
|  | **Scope** | | | |  | **Enablers** | |  | **Population health** | | | | |  | **Experience of care** | |  | **Cost and utilization** | |
|  | Clinical level | Professional level | Organisational level | System level |  | Functional | Normative |  | Mortality | Morbidity | Disease burden | Behavioural factors | Physiological factors |  | Satisfaction | Quality of care |  | Costs per capita | Utilization of services |
| Jiamjariyaporn et al. (2014) [64] | • | • |  |  |  |  |  |  |  |  |  | • | • |  |  |  |  |  |  |
| Blickem et al. (2013) [65] | • |  |  |  |  |  |  |  |  | • |  |  |  |  |  |  |  | • | • |
| Spiegel et al. (2010) [66] | • | • |  |  |  | • | • |  | • |  |  |  |  |  |  |  |  |  |  |
| Blake (2001) [67] | • |  |  |  |  |  |  |  | • |  | • |  |  |  |  | • |  | • |  |
| Van Biesen et al. (2000) [68] | • |  |  |  |  |  |  |  |  |  |  |  | • |  |  |  |  |  |  |
| Maddux et al. (2013) [69] |  |  | • |  |  | • |  |  | • | • |  |  |  |  |  |  |  | • | • |
| Pai et al. (2013) [70] | • | • |  |  |  | • |  |  | • |  |  |  | • |  |  | • |  | • | • |
| Ramos & Molina (2013) [71] | • | • |  |  |  | • |  |  | • | • |  |  |  |  |  | • |  |  | • |
| Norfolk & Hartle (2013) [72] | • | • | • |  |  | • |  |  | • | • |  |  | • |  |  |  |  | • |  |
| Bowman et al. (2013) [73] | • | • |  |  |  | • | • |  | • |  |  | • | • |  |  | • |  |  |  |
| Harden et al. (2012) [74] | • | • |  |  |  |  |  |  |  |  |  | • |  |  |  | • |  |  | • |
| Clancy (2011) [75] |  | • |  |  |  | • |  |  |  |  |  |  | • |  | • | • |  |  |  |
| van der Veer et al. (2011) [76] | • | • |  |  |  | • |  |  |  |  |  | • | • |  |  | • |  |  |  |
| Virgilio (2010) [77] |  | • |  |  |  | • |  |  | • |  |  |  | • |  |  |  |  |  | • |
| Davison (2010) [78] | • |  |  |  |  |  |  |  |  |  |  |  | • |  | • |  |  |  |  |
| Finkelstein et al. (2009) [79] | • |  |  |  |  |  |  |  |  | • |  | • |  |  |  |  |  |  |  |
| Almaguer et al. (2006) [80] | • | • |  |  |  |  |  |  |  |  | • | • | • |  |  |  |  |  |  |
| Owen et al. (2006) [81] | • | • |  |  |  |  |  |  |  |  | • |  | • |  |  | • |  |  |  |
| Crooks (2004) [82] | • | • | • |  |  | • |  |  |  | • | • |  |  |  |  |  |  | • | • |
| Lindsay et al. (2006) [83] | • |  |  |  |  |  |  |  |  | • |  | • |  |  |  | • |  |  |  |
| Smith et al. (2004) [84] |  |  | • |  |  |  |  |  |  | • |  |  |  |  |  |  |  | • | • |
| Fischer et al. (2011) [29] | • | • |  | • |  |  |  |  |  |  | • |  | • |  |  |  |  |  |  |
| Suri et al. (2007) [85] | • |  |  |  |  |  |  |  |  |  | • |  | • |  |  | • |  | • | • |
| Humphreys et al. (2012) [86] | | • | • |  |  | • |  |  |  |  | • |  |  |  |  |  |  |  |  |
| Manns et al. (2010) [87] |  |  |  |  |  | • |  |  | • | • | • |  | • |  |  |  |  | • |  |
| Keith et al. (2004) [88] | • |  | • |  |  |  |  |  | • | • | • |  | • |  |  |  |  |  |  |
| Rognant et al. (2013) [89] | • | • |  |  |  |  |  |  |  |  |  | • | • |  |  |  |  |  | • |
| Lee et al. (2009) [90] |  |  |  |  |  | • |  |  |  | • |  |  |  |  |  |  |  | • | • |
| Hamm et al. (2013) [91] | • | • |  |  |  | • |  |  |  |  |  |  |  |  |  | • |  |  |  |
| Bayliss et al. (2011) [92] | • | • | • |  |  |  |  |  |  |  | • | • | • |  |  |  |  |  |  |
| Barahimi et al. (2014) [93] | • | • |  |  |  |  |  |  |  |  |  |  | • |  |  |  |  |  |  |
| Schachter et al. (2013) [94] | | • |  |  |  | • |  |  |  |  |  |  | • |  |  | • |  |  |  |
| Vachharajani et al. (2010) [95] | • | • |  |  |  |  |  |  |  |  |  |  | • |  |  | • |  |  |  |
